# Muscle Microbiopsy to Delineate Stem Cell Involvement in Young Patients: A Novel Approach for Children With Cerebral Palsy

**DOI:** 10.3389/fphys.2020.00945

**Published:** 2020-08-06

**Authors:** Marlies Corvelyn, Nathalie De Beukelaer, Robin Duelen, Jorieke Deschrevel, Anja Van Campenhout, Sandra Prinsen, Ghislaine Gayan-Ramirez, Karen Maes, Guido Weide, Kaat Desloovere, Maurilio Sampaolesi, Domiziana Costamagna

**Affiliations:** ^1^Stem Cell Biology and Embryology, Department of Development and Regeneration, KU Leuven, Leuven, Belgium; ^2^Neurorehabilitation Group, Department of Rehabilitation Sciences, KU Leuven, Leuven, Belgium; ^3^Laboratory of Respiratory Disease and Thoracic Surgery, Department of Chronic Diseases and Metabolism, KU Leuven, Leuven, Belgium; ^4^Pediatric Orthopedics, Department of Development and Regeneration, KU Leuven, Leuven, Belgium

**Keywords:** cerebral palsy, young children, muscle microbiopsy, adult muscle stem cell-derived progenitors, differentiation potential, myogenesis

## Abstract

Cerebral palsy (CP), the single largest cause of childhood physical disability, is characterized firstly by a lesion in the immature brain, and secondly by musculoskeletal problems that progress with age. Previous research reported altered muscle properties, such as reduced volume and satellite cell (SC) numbers and hypertrophic extracellular matrix compared to typically developing (TD) children (>10 years). Unfortunately, data on younger CP patients are scarce and studies on SCs and other muscle stem cells in CP are insufficient or lacking. Therefore, it remains difficult to understand the early onset and trajectory of altered muscle properties in growing CP children. Because muscle stem cells are responsible for postnatal growth, repair and remodeling, multiple adult stem cell populations from young CP children could play a role in altered muscle development. To this end, new methods for studying muscle samples of young children, valid to delineate the features and to elucidate the regenerative potential of muscle tissue, are necessary. Using minimal invasive muscle microbiopsy, which was applied in young subjects under general anaesthesia for the first time, we aimed to isolate and characterize muscle stem cell-derived progenitors of TD children and patients with CP. Data of 15 CP patients, 3–9 years old, and 5 aged-matched TD children were reported. The muscle microbiopsy technique was tolerated well in all participants. Through the explant technique, we provided muscle stem cell-derived progenitors from the *Medial Gastrocnemius.* Via fluorescent activated cell sorting, using surface markers CD56, ALP, and PDGFRa, we obtained SC-derived progenitors, mesoangioblasts and fibro-adipogenic progenitors, respectively. Adipogenic, skeletal, and smooth muscle differentiation assays confirmed the cell identity and ability to give rise to different cell types after appropriate stimuli. Myogenic differentiation in CP SC-derived progenitors showed enhanced fusion index and altered myotube formation based on MYOSIN HEAVY CHAIN expression, as well as disorganization of nuclear spreading, which were not observed in TD myotubes. In conclusion, the microbiopsy technique allows more focused muscle research in young CP patients. Current results show altered differentiation abilities of muscle stem cell-derived progenitors and support the hypothesis of their involvement in CP-altered muscle growth.

## Introduction

Cerebral palsy (CP) is the single largest lifelong condition leading to childhood physical disability, affecting 1 in 500 newborns (Christensen et al., 2014). It is characterized firstly by neural deficits caused by a non-progressive lesion in the immature brain, and secondly by musculoskeletal problems that progress with age (Mathewson and Lieber, 2015). This condition manifests itself through alterations in motor development and function such as loss of selective motor control. Clinical symptoms of the neural and muscular impairments mainly involve spasticity, increased stiffness and contractures, muscle weakness and decreased functional ability such as disturbed gait (Kurz et al., 2012). Based on their functional capacities, patients with CP are subdivided following Gross Motor Function Classification System (GMFCS) levels (Palisano et al., 2008). Although the initial problem is neural in origin, most treatments are directed at muscle improvements such as physiotherapy, casting, orthoses and botulinum toxin (BTX) injections (Williams et al., 2013; Mathewson and Lieber, 2015). Not only heterogeneity in clinical representation, but also in etiology, i.e., timing and cause of the brain injury complicate research and no single animal model has been sufficient to recapitulate all aspects of motor dysfunction in children with CP (Cavarsan et al., 2019).

Muscle alterations have been observed in comparison to typically developing (TD) children both at macroscopic (e.g., reduced muscle volume and longer tendons; Booth et al., 2001; Foran et al., 2005) and microscopic levels (e.g., fibrotic tissue accumulation, hypertrophic extracellular matrix and reduced satellite cell (SC) numbers; Dayanidhi et al., 2015). These alterations play an important role in the pathology and clinical representation of CP. For example, higher collagen content in the interstitium of the muscle has been associated with higher stiffness (Smith et al., 2019). On the other hand, SC ablation in a transgenic mouse model showed a reduced serial sarcomere number and low recovery ability after immobilization. These features could explain the difficulties that children with CP experience in recovering from an immobilization-induced contracture. Moreover, in these patients, the low addition of serial sarcomere number could be due to a decreased number of SCs (Dayanidhi et al., 2020). Unfortunately, the onset and development of these muscle alterations are not well understood, because data from young patients with CP, i.e., <10 years of age, are still unavailable. Studies examining the microscopic muscle features of children with CP are based on biopsy samples that are mainly obtained during invasive surgery. Usually, surgical correction is conducted in a subgroup of the patient population, commonly at an age over 8–10 years. The timing of these interventions is not appropriate to associate muscle biopsies able to unravel the onset of muscle alterations in CP. Hence, the use of less invasive techniques to collect muscle biopsies earlier in the course of the disease is preferable and essential for optimizing treatment (Booth et al., 2001; Marbini et al., 2002; Domenighetti et al., 2018).

Skeletal muscle is a very dynamic tissue characterized, firstly, by postnatal development and, secondly, by remodeling and repair processes occurring during life, i.e., in response to growth, exercise, injury and surgery (Dreyer et al., 2006; Fu et al., 2015; Snijders et al., 2015). The most well-known muscle progenitors are SCs that reside between the sarcolemma and the basal lamina of the skeletal muscle. These cells play a crucial role in postnatal muscle development as extensively described, mostly in mice (White et al., 2010; Neal et al., 2012). During postnatal development longitudinal and radial muscle fiber growth has been reported in mice (Gokhin et al., 2008). Fusion of the myoblasts from SCs into existing myofibers induces a twofold increase in myofibrillar packing, sevenfold enlargement in myofiber cross-sectional area, with consequent fourfold growth of muscle mass (Gokhin et al., 2008; Dayanidhi and Lieber, 2014). Myofiber longitudinal length increases with addition of sarcomeres-in-series during the first 4–6 postnatal weeks (Griffin et al., 1971). Moreover, overload-induced hypertrophy depends on SC availability and on maturational age, because the overall response to overload differs in young versus mature mice (Murach et al., 2017). These SCs respond to external triggers such as atrophic and anabolic stimuli, hormones and cytokines, typically after acute and chronic inflammation (Costamagna et al., 2015). These adult stem cells are normally in a quiescent state but, due to environmental cues, such as muscle injury, exercise, eccentric strength as other triggers, they become activated (Dreyer et al., 2006; Fu et al., 2015; Snijders et al., 2015). Once activated, SCs start to proliferate and either self-renew for maintaining the muscle stem cell pool or differentiate toward myoblasts and fuse with existing myofibers to increase fiber mass and length (Tidball, 2011). During chronic inflammation conditions, SC number can increase due to inflammatory stimuli, such as those in muscular dystrophies, where the number of SCs is eventually exhausted, or during cancer-induced muscle atrophy, where SCs accumulate in the interstitium (He et al., 2013; Costamagna et al., 2020). Despite their specific location, SCs can be identified by different molecular markers such as PAX7, CD56, and CD82 (Illa et al., 1992; Seale et al., 2000; Tedesco et al., 2017; Rotini et al., 2018; Tey et al., 2019). Activation markers of SCs include NCAM (CD56), MyoD, Dlk1, cMet, Myf5 etc. (Lindström et al., 2010; Snijders et al., 2015).

Previous studies on adolescents showed that the number of SCs in contractured muscles of patients with CP is decreased in comparison to TD controls (Smith et al., 2013; Dayanidhi et al., 2015; Von Walden et al., 2018; Smith et al., 2019). As SCs are considered to be the source of myonuclei, and the number of nuclei was not found to be altered in CP adolescents, it has been suggested that the reduction in SCs is unlikely to occur at a very young age when different myoblasts fuse with multinucleated fibers (Dayanidhi et al., 2015). However, this hypothesis still needs to be tested. Additionally, recent *in vitro* studies showed a decreased myogenic capacity for differentiation and fusion of these SCs in CP adolescents (Domenighetti et al., 2018). The role and the involvement for multiple muscle precursors at young ages in patients with CP are currently unknown.

The current knowledge regarding muscle regeneration potential in young CP muscle needs to be broadened by determining whether other cell types that can play a role in muscle regeneration are affected. As such mesoangioblasts (MABs) and fibro-adipogenic progenitors (FAPs) in particular have been recognized for their role in regeneration processes for either direct fusion into myofibers (MABs) or a supportive role (FAPs) toward other cell types, such as SCs (Bentzinger et al., 2013; Ceafalan et al., 2014). MABs are multipotent progenitors able to give rise to extravascular mesodermal cell types such as smooth, cardiac and skeletal muscle, bone cells and adipocytes *in vitro* (Tonlorenzi et al., 2007; Messina et al., 2009; Quattrocelli et al., 2012). These cells withhold great therapeutic interest, because they can migrate through the blood vessels (Klimczak et al., 2018). Moreover, MABs have been shown to be able to contribute to the SC pool in case the SCs are exhausted or insufficient for muscle regeneration and repair as in case of muscular dystrophies (Tedesco et al., 2017). In this respect, promising results have been obtained in preclinical studies applying MAB-based treatments for dystrophic experimental conditions (Sampaolesi et al., 2003, 2006; Bosurgi et al., 2015). A few years ago, safety of MAB systematic injections in patients with Duchenne muscular dystrophy was successfully proved and further studies are shortly expected to show MAB efficacy in contributing to the contractile material of dystrophic patients (Cossu et al., 2015).

In addition to MABs, FAPs have also been isolated from the muscle interstitium and have been thoroughly described in mice (Joe et al., 2010; Uezumi et al., 2010). Murine FAPs do not directly fuse with damaged muscle fibers. However, upon injury, FAPs are attracted by inflammatory cytokines, start to proliferate under the impulse of IL-4/IL-13 secretion of eosinophils and support myogenesis, enhancing the differentiation through signaling molecules (Heredia et al., 2013). Under pathologic conditions, FAPs can adopt an adipogenic cell fate for which the mechanisms are still poorly understood (Judson et al., 2017). Although FAPs still need to be carefully identified in young human subjects, some groups, included ours, have tried to isolate them from human samples through the expression of specific surface markers such as CD15 and PDGFRa (Bentzinger et al., 2013; Uezumi et al., 2014; Arrighi et al., 2015; Rotini et al., 2018). However, the knowledge regarding their potential role in muscle regeneration and repair processes in humans is still limited. Due to their indirect effects on regeneration and involvement in fibro-adipogenesis, these cells might be of great interest for their role in multiple muscular pathologies (Heredia et al., 2013; Tedesco et al., 2017). Further studies are needed to explore new markers able to recognize the corresponding functional murine population in human adult muscle.

Spurred by the importance of muscle stem cells and the lack of information in altered CP muscles, the current study investigates the feasibility of the muscle microbiopsy technique for the systematic isolation of adult stem cell-derived progenitors, as this technique already showed many advantages in previous applications (Magistris et al., 1998; Hayot et al., 2005; Pomiès et al., 2015; Townsend et al., 2016). Because this technique is minimally invasive, well-tolerated and harmless, it is considered ideal to be applied in young children. We specifically focused on SC-derived progenitors, MABs and FAPs in view of their potentially coordinated and supportive role in muscle development and regeneration as described earlier, especially in situations of insufficient SCs, as it is the case for CP muscles. Multiple stem cell-derived progenitors were isolated by fluorescent activated cell sorting (FACS), characterized and assessed for their specific differentiation abilities *in vitro* (Marg et al., 2014) (as shown in the additional Figure 1 in section “Materials and Methods”). We reported higher fusion index values of SC-derived progenitors from patients with CP after six days of myogenic differentiation in comparison to those of TD children. No differences were described for MABs and FAPs- populations between TD children and patients with CP. Finally, we highlighted important features as the focus for future CP studies and pointed out myotube morphology alterations that seemed to characterize CP SC-derived progenitors.

**FIGURE 1:**
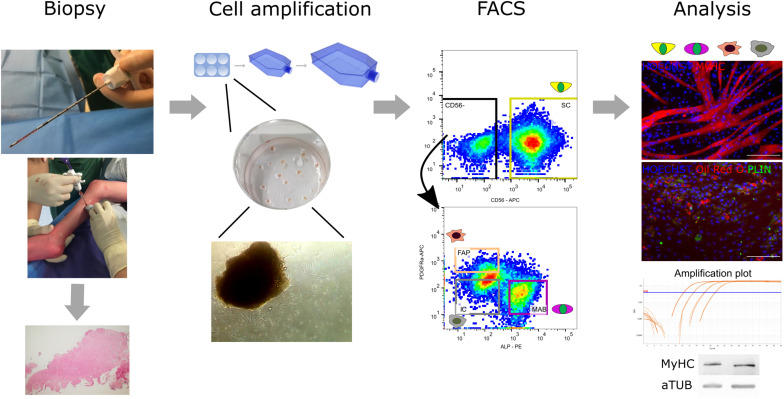
Flowchart from muscle microbiopsy collection to cellular analyses. From left to right: the muscle microbiopsies were collected from the *Medial Gastrocnemius* under ultrasound guidance from both TD children and patients with CP. Microbiopsies were either cut and histologically assessed or minced and cultured on collagen-coated plates to isolate extruding cells able to proliferate further. After amplification of the cells, a first fluorescent activated cell sorting (FACS) at passage 3–4 was performed based on CD56, a commonly accepted marker for satellite cell (SC)-derived progenitors. Subsequently, around passage 6–8, the CD56^–^ population underwent a second FACS based on markers from literature ALP and PDGFRa for isolation of mesoangioblasts (MABs) and fibro-adipogenic progenitor cells (FAPs), respectively. Together with the CD56^–^ ALP^–^ PDGFRa^–^ population (ICs), all obtained cell types were assessed for their differentiation potential and harvested for immunofluorescence, RT-qPCR and western blot analysis (MyHC, MYOSIN HEAVY CHAIN; PLIN, PERILIPIN; aTUB, α TUBULIN).

## Materials and Methods

### Biopsy Collection

This study protocol was approved by the Ethical Committee of the University Hospitals of Leuven, Belgium (S61110 and S62645). Written informed consent was obtained from the parents. Children with CP were recruited from the CP Reference Centre, whereas TD children were recruited from the Traumatology Unit of the University Hospitals Leuven (Belgium). A group of 15 children with uni- or bilateral Cerebral Palsy (CP; aged 6.3 ± 2.0; range 3.1–9.0 years) with Gross Motor Function Classification System (GMFCS) (Palisano et al., 2008) level I-III and 5 aged-matched TD children (aged 5.1 ± 1.4; range: 3.4–7.2 years) were included in this study. In the CP group, children with presence of dystonia or ataxia, BTX injections within the last 6 months, orthopedic surgery less than 2 years before as well as any muscle surgery on the included muscles were excluded. Additionally, TD children were further excluded when they had a history of neurological problems or when they were involved in a high-performance sporting program. Demographic and anthropometric characteristics for the recruited children are represented in Table 1. No significant differences were found in age, body mass and height between the CP and TD groups. All patients wore orthoses and underwent regular physiotherapy as part of their standard of care. Muscle microbiopsies were obtained from the muscle belly of the *Medial Gastrocnemius* during interventions requiring general anesthesia (BTX injections or orthopedic surgery) for patients with CP. In TD children, microbiopsies were obtained while material from upper limb trauma surgery was removed. The biopsy collections were performed percutaneously, under ultrasound guidance with a microbiopsy needle (16-gauge, Bard). The acquired microbiopsies had a maximum length of 1.5 cm, weighed on average 10 ± 2.2 mg for TD and 9.5 ± 2.4 mg for CP and had a diameter of 1.3 mm. No significant differences were present in the weight of the microbiopsies between the groups. Immediately after isolation, muscle microbiopsies were divided and disrupted in smaller pieces. These were cultured as explained in the “Materials and Methods” section and shown in the reported flowchart (Figure 1). After the biopsy collection, ice packs were used to avoid hematoma. Clinical tolerance was good and the parents were asked to evaluate the associated pain during the days following the biopsy procedure. Among all subjects, few complained about discomfort, stiffness and minor pain during the 2 days after biopsy collection. After 2 days no discomfort was reported in any subjects.

**TABLE 1:** Demographic and anthropometric data for the recruited subjects.

		**TD (*n* = 5)**	**CP (*n* = 15)**			

				**GMFCS I (*n* = 5)**	**GMFCS II (*n* = 7)**	**GMFCS III (*n* = 3)**
Age (year)	Mean (*SD*)	5.1 (1.4)	6.3 (2.0)	5.2 (1.7)	6.1 (2.0)	8.5 (0.4)
	Range	3.4–7.2	3.1–8.9	3.1–7.5	3.7–8.9	8.0–8.9
Sex (M – F)	N	2–3	10–5			
Body mass (KG)	Mean (*SD*)	18.8 (3.7)	20.9 (6.1)	17.4 (2.8)	19.5 (4.6)	29.9 (4.9)
Height (CM)	Mean (*SD*)	110.9 (9.3)	114.2 (15.6)	104.5 (10.4)	113.6 (15.5)	131.7 (9.1)
Involvement (unilat – bilat)	N	–	5–10	3–2	2–5	0–3
Number of previous btx injections (N)	Mean (*SD*)	–	1.7 (2.2)	1.4 (1.3)	2.3 (3.0)	1.0 (1.0)
	Range	–	0–7	0–3	0–7	0–2
Physiotherapy (min/week)	Mean (*SD*)	–	139.7 (60.1)	123.0 (60.4)	158.6 (56.7)	123.3 (77.7)

**In this study, two subject groups were included: typically developing (TD) children and patients with Cerebral Palsy (CP). Both groups did not differ in age, body, mass and height. Patients with CP are further subdivided based on Gross Motor Function Classification System (GMFCS) levels. Phenotype was described by unilateral or bilateral involvement. Number of previous botulinum toxin (BTX) injections in the Medial Gastrocnemius is reported. Mean and standard deviation (SD) are shown. Unpaired T-tests were performed to compare the two groups (TD and CP children).**

### Cell Culture

Muscle biopsies were cultured in multi-well dishes coated with 0.1% bovine collagen (Sigma-Aldrich for all of the compounds, as declared elsewhere) at 37°C in a 5% CO_2_ and 5% O_2_ atmosphere according to the explant technique (Cusella-De Angelis et al., 1994; Messina et al., 2004; Morosetti et al., 2006; Tonlorenzi et al., 2007; Díaz-Manera et al., 2012; Quattrocelli et al., 2012), as direct enzymatic digestion of the microbiopsy resulted in too low yields for stem cell-derived progenitor isolation, culture and further experiments. Growth medium containing IMDM (Thermo Fisher Scientific) was used with 20% fetal bovine serum (FBS; Thermo Fisher Scientific), 1 mg/mL non-essential aminoacids (Thermo Fisher Scientific), 1 mg/mL sodium-pyruvate (Thermo Fisher Scientific), 1 U/mL penicillin/streptomycin (P/S; Thermo Fisher Scientific), 1% chicken embryo extract (CEE; Bio Connect), 2 mM Glutamine (Thermo Fisher Scientific) and 100 nM β-mercaptoethanol (Thermo Fisher Scientific). When 70–80% of confluence was reached, cells were passaged, using TrypLE^TM^ Express (Thermo Fisher Scientific). After cell sorting and amplification, cells were seeded at different densities to test their differentiation potential using specific differentiation media to induce different cell fate programs, as specified in “*In vitro* differentiation assays.”

### Fluorescent Activated Cell Sorting

Cell populations were isolated by serial FACS with a BD FACSAria II (BD biosciences) device. The first FACS was performed on cells at passage 3-4 (P3-P4), the second at P6-P8. Three to 10 × 10^6^ cells, once detached, were serial sorted first with a commonly accepted marker for satellite cells (SC-derived progenitors; CD56) (Illa et al., 1992; Mackey et al., 2007; Lecourt et al., 2010; Scott et al., 2013; Snijders et al., 2015; Pomiès et al., 2015) and in a following sorting on the CD56^–^ population, with a mesoangioblast marker (MAB; Alkaline Phosphatase, ALP) (Dellavalle et al., 2007; Crippa et al., 2011; Díaz-Manera et al., 2012; Quattrocelli et al., 2012) and a fibro-adipogenic progenitor cell marker (FAP; Platelet-Derived-Growth Factor Receptor alpha; PDGFRa and CD15) (Uezumi et al., 2014; Arrighi et al., 2015; Rotini et al., 2018) as specified in Table 3. Cells were incubated with the primary antibodies at room temperature (RT) for 30 min, protected from light. FACS cell gating prior to the sorting was performed through forward and side scatter plots to gate the main population of interest, excluding non-viable cells based on dimension and granularity. Further gates were selected for single cells. Finally, to assess correct gating, bare cells and negative controls (fluorescent minus one, FMO) were used. A fraction of the CD56^–^ ALP^–^ PDGFRa^–^ population was also isolated. After sorting, the cells were seeded at densities of over 1 × 10^4^ cells/cm^2^. Analyses were performed using FACS DIVA software. Because six of the included patients with CP were initially enrolled in a pilot study (S61110) for optimization of this workflow, not all data from these patients could be included in all analyses.

### Flow Cytometry

Antibody titrations for FACS optimization and further flow cytometry analyses were performed with a FACSCanto II AIG (BD biosciences). For ALP and PDGFRa titrations, antibody concentrations within the range of 15 ng/mL to 2 μg/mL were used. To assess correct gating, 1 × 10^6^ cells were used as bare cells or negative controls (FMOs). Analyses were performed using FlowJo v10.6.1 software.

### *In vitro* Differentiation Assays

All differentiations were conducted in the same conditions as for cultivation, namely at 37°C in a 5% CO_2_ and 5% O_2_ atmosphere. For both skeletal muscle and adipocyte differentiation, cells were seeded at 6 × 10^4^ cells/cm^2^ in multi-well dishes and cultured with growth medium. For myogenic induction, when cells were at 80% of their confluence, differentiation medium was applied. Myogenic differentiation medium consisted of DMEM High Glucose (Thermo Fisher Scientific), 2% horse serum (Thermo Fisher Scientific), 1% CEE, 1 mg/mL sodium-pyruvate and 1 U/mL P/S. Medium was replaced every other day and myogenic differentiation was terminated after 3 and 6 days for all samples. For adipogenic differentiation, the StemPro Adipogenesis Differentiation Medium was applied when the seeded cells reached almost 100% confluence, following the density and the conditions advised by the provider (Thermo Fisher Scientific). Smooth muscle differentiation was performed at a lower confluence of 3 × 10^3^ cells/cm^2^, replacing the medium the day after seeding by myogenic differentiation medium supplemented with 50 ng/mL TGFb1 (Peprotech). For all adipogenic and smooth muscle differentiations, the medium was replaced every other day and the differentiation was terminated after 10 days.

### Quantitative RT-PCR Pursuing Myogenic Fusion and Differentiation

RNA isolation was carried out using the PureLink^®^ RNA Mini Kit (Thermo Fisher Scientific) according to the provided protocol. Genomic DNA traces were removed using Turbo DNase (Thermo Fisher Scientific), following the manufacturer’s instructions. RNA concentration was quantified by Spectrophotometer ND-1000 (Nanodrop) and integrity was assessed by agarose gel electrophoresis. First strand cDNA synthesis was carried out based on 200 ng of total RNA (Vandesompele et al., 2002; Arning et al., 2004; Pomiès et al., 2016; Von Walden et al., 2018) following the protocol provided by the Superscript III First-Strand Synthesis SuperMix for RT-qPCR kit (Thermo Fisher Scientific). RT-qPCR reaction mix consisted of 1:5 diluted cDNA (in Milli-Q water, MQ), one volume of SYBR green (Qiagen), 25 nM ROX (Thermo Fisher Scientific) and 0.25 μM primers (Table 2). The analysis was carried out in 384 well plates with the ViiA^TM^ 7 Real-Time PCR system (Thermo Fisher Scientific). The run method consisted of 95°C for 20 s, 40 cycles of 95°C for 1 s and 60°C cycles for 20 s followed by melting curve analysis. Gene expression levels were normalized to housekeeping gene β*-ACTIN*. dCT was obtained by subtracting the CT of the investigated gene from the CT of the housekeeping gene.

**TABLE 2:** Primer list for RT-qPCR.

**Gene**	**Forward primer**	**Reverse primer**
*PAX7*	GGGCCTCCTGCTTGTTTAT	CCATCTGGCTGGACTTCAAT
*MYOD*	CCGCCTGAGCAAAGTAAATG	CGATATAGCGGATGGCGTT
*MyHC*	GACATTGACCACACCCAGTATAA	CAGCTTCTCATCTCGCATCTC
*MYOMAKER*	TCATCATCGCGGCAAAGT	AGCAGCAGAACAAAGGACATA
*DESMIN*	GAAGCTGCTGGAGGGAGAG	ATGGACCTCAGAACCCCTTT
*KIF5b*	AGGAAGCAGTCAGGTCAAAG	CACTTGGGTGAGTTGGAGAA
*ITGB1*	TGATCCTGTGTCCCATTGTAAG	GCAGTAATGCAAGGCCAATAAG
β*-ACTIN*	GGACCTGACTGACTACCTCAT	CGTAGCACAGCTTCTCCTTAAT

### Immunofluorescent Staining

Cells were cultured in 96 well dishes (Thermo Fisher Scientific) and fixed with 4% paraformaldehyde. Samples were incubated at RT in permeabilization medium (1% Bovine Serum Albumin, 0.2% TritonX-100 in Phosphate Buffer Solution, PBS), for 30 min, followed by blocking with 1:10 donkey serum (VWR) in PBS, at RT for 30 min. Cells were incubated overnight with primary antibody at 4°C. A list of the used antibodies is provided in Table 3. Before applying the appropriate secondary antibody (1:500, Alexa Fluor^®^ donkey 488 or 594, Thermo Fisher Scientific), the cells were washed twice with PBS. Hoechst (1:3000 in PBS, Thermo Fisher Scientific) was added for 1 min and the cells were washed twice with PBS. For fusion index (ratio between myonuclei and total number of nuclei in a field), we counted the nuclei in MYOSIN HEAVY CHAIN (MyHC) positive myotubes composed of at least 2 nuclei. This was assessed in 3 randomized pictures for every well in pictures taken at 10x magnification. Visualization occurred with an Eclipse Ti Microscope (Nikon) and NIS-Elements AR 4.11 Software.

**TABLE 3:** List of antibodies for FACS, immunofluorescent staining and western blot analyses.

**Antibody**	**Relative dilution**	**Brand**	**Application**
CD56-APC	0.5 μL/1 × 10^6^ cells	BioLegend	FACS
Alkaline phospatase-PE	3.0 μL/1 × 10^6^ cells	R&D Systems	FACS
PDGFRa-APC	2.5 μL/1 × 10^6^ cells	BioLegend	FACS
CD15-FITC	10 μL/1 × 10^6^ cells	BD Pharmingen	FACS
CD56 (1)	1:1000 (mouse)	BD Pharmingen	Immunofluorescence (muscle slide)
(2)	1:300 (mouse)		Immunofluorescence (*in vitro* staining)
PERILIPIN A/B	1:200 (rabbit)	Sigma-Aldrich	Immunofluorescence
MF20	1:20 (mouse)	Hybridoma Bank	Immunofluorescence
MYOD	1:200 (rabbit)	Cell Signaling	Immunofluorescence
α Smooth muscle actin	1:300 (mouse)	Sigma-Aldrich	Immunofluorescence
CD90	1:100 (rabbit)	Abcam	Immunofluorescence
PAX7	1:2 (mouse)	Hybridoma Bank	Immunofluorescence
LAMININ	1:250 (rabbit)	Abcam	Immunofluorescence
MF20	1:4 (mouse)	Hybridoma Bank	Western blot
α TUBULIN	1:1000 (mouse)	Sigma-Aldrich	Western blot

### Oil Red O Staining to Assess Deposition of Lipid Droplets

For adipogenic differentiation, Oil Red O staining was performed. Cells were washed twice with MQ water and incubated with Oil Red O solution (65% of 0.5% w/v Oil Red O in isopropanol (Thermo Fisher Scientific) and MQ water) for 50 min. Cells were washed extensively with MQ water before carrying out the previously described standard immunofluorescence protocol for visualization of the adipocyte membrane with PERILIPIN (PLIN; Table 3). To quantify Oil Red O staining, lipids were extracted with a petrol ether/isopropanol mixture (3:2) and quantified for their absorbance at 490 nm for 0.1 s with Victor Spectrophotometer (PerkinElmer). Standard curve was applied and quantification expressed as a percentage of the absorbance observed in TD samples.

### Western Blot to Quantify MyHC Levels in Differentiated Satellite Cells

Cells were resuspended in 60 μL of RIPA buffer supplemented with 10 mM sodium fluoride, 0.5 mM sodium orthovanadate, 1:100 protease inhibitor cocktail and 1 mM phenylmethanesulfonyl fluoride. The cells were disrupted using a 21-gauge needle, kept on ice for 30 min, sonicated and afterwards incubated on ice for 30 min. Samples were centrifuged at 10,000 g for 10 min at 4°C. The supernatant was transferred into a new tube and protein concentrations were measured by a Spectrophotometer ND-1000 (Nanodrop). 40 μg of proteins were heat-denatured in sample-loading buffer (50 mM tris-HCl, pH 6.8, 100 mM DTT, 2% SDS, 0.1% bromophenol blue, 10% glycerol) at 95°C for 10 min. Proteins were separated by a 10% SDS-polyacrylamide gel electrophoresis and transferred to nitrocellulose membranes. Blocking was performed using 5% non-fat dry milk dissolved in tris-buffered saline (TBS) containing 0.05% Tween. Incubation with primary antibody was performed overnight in TBS-Tween with 2.5% milk. Secondary horseradish peroxidase (HRP)-conjugated antibody (BioRad) was diluted 1:5000 in TBS-Tween with 2.5% milk. Protein amounts were normalized using a mouse antibody against α-TUBULIN. Western blot analysis was performed with a GelDoc chemioluminescence detection system (Bio-Rad). Quantitation was performed and relative densitometry was calculated by normalizing the protein band against the background and housekeeping proteins, using the QuantityOne software (BioRad).

### Histological Analyses

Muscle microbiopsies were frozen in isopentane cooled in liquid nitrogen and stored at −80°C for further analyses. Five μm muscle sections were obtained using a CryoStar^TM^ NX70 Cryostat (Thermo Fisher Scientific), while kept at −20°C. Sections were brought to room temperature for 10 min in a humid chamber then incubated in cold acetone for 10 min. After washing with PBS, slides were incubated with 10% goat serum (Invitrogen) for 1 h. Primary antibodies at different dilutions were applied overnight at 4°C as reported in Table 3. After extensive washing with PBS, muscle slides were incubated for 1 h at RT with appropriate secondary antibodies (1:500, Alexa Fluor^®^ goat 488, 555 or 680, Thermo Fisher Scientific). Dapi (1:50 in PBS, Thermo Fisher Scientific) was added for 1 min and the samples were extensively washed with PBS before mounting with ProLong^®^ Gold antifade reagent (Molecular Probes). Visualization was performed using an inverted DMi8 microscope (Leica) using LasX software.

### Statistics

The patients in this study showed a normal distribution based on age, length and body weight. Hence, results are expressed as mean values with standard deviation (SD). Due to the limited sample sizes, boxplots were used in the visualization of the fusion index values and of Oil Red O quantifications. The boxplots indicate the 25th to 75th percentiles, while the whiskers indicate the minimum and maximum values. The center of the boxplot indicates the median. Unpaired *T*-tests were performed for comparison between the TD and CP groups (two-tailed, *p* < 0.05). One-way ANOVA was performed to compare the subgroups of children with CP characterized by the same GMFCS levels or BTX treatment history. Comparison of multiple subgroups and time points for RT-qPCR were assessed through two-way ANOVA, followed by Tukey’s multiple comparison tests. Significance of the differences was reported as ^∗^*p* < 0.05. Statistical analysis was performed with GraphPad Prism 8 software. Regression analysis was performed with Excel (Office 2019). Power analysis was performed using G Power 3.1.9.7.

## Results

The procedure of collecting muscle biopsy through the muscle microbiopsy technique was well-tolerated and provided enough material to be plated and amplified on collagen coated dishes. The complete workflow was performed starting from FACS analysis to obtain stem cell-derived progenitors from first satellite cells (SCs) and subsequently mesoangioblasts (MABs), fibro-adipogenic progenitors (FAPs) and the CD56^–^ ALP^–^ PDGFRa^–^ populations (see Figure 1). The potential of these cells was evaluated based on myogenic differentiation for SC-derived progenitors; skeletal muscle, adipogenic and smooth muscle differentiation for MABs; myogenic and adipogenic differentiation for FAPs and interstitial cell (IC; CD56^–^ ALP^–^ PDGFRa^–^) populations. Because a number of the subjects included was enrolled in a pilot study (S61110) for optimization of this workflow, not all the analyses could be performed in all CP samples. The numbers of subjects are specified for each analysis.

### Adult Stem Cell Histological Identification and Isolation

**FIGURE 2:**
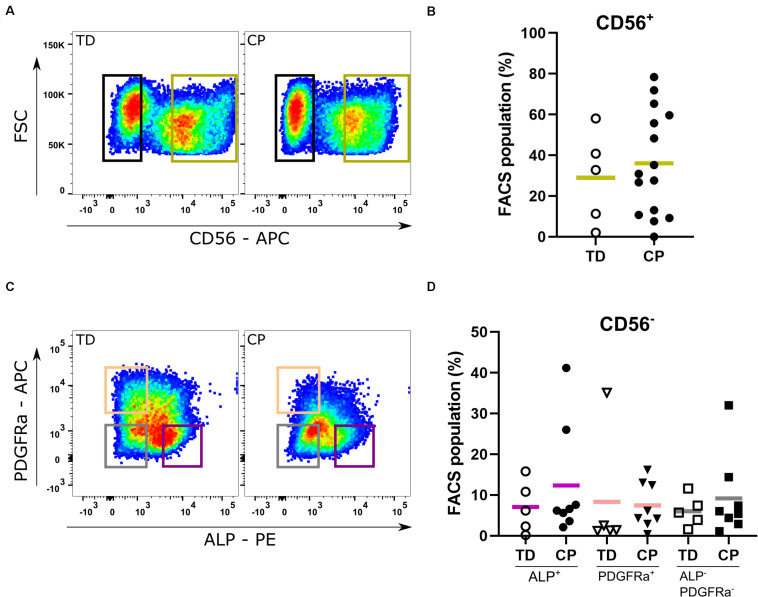
Fluorescent Activated Cell Sorting of muscle stem cells. **(A)** Example of Fluorescent Activated Cell Sorting (FACS): based on the expression of CD56, satellite cell-derived progenitors (SCs; green gate) were isolated from both TD children (*n* = 5) as well as patients with CP (*n* = 15). **(B)** Percentages of CD56^+^ cells from each child are reported in the graph. **(C)** Example of FACS: after amplification, FACS on the CD56^–^ population isolated mesoangioblasts (MABs; CD56^–^ PDGFRa^–^ ALP^+^; purple gate), fibro-adipogenic progenitors (FAPs; CD56^–^ ALP^–^ PDGFRa^+^; orange gate) and interstitial cells (ICs; CD56^–^ ALP^–^ PDGFRa^–^; gray gate) (TD: *n* = 5; CP: *n* = 8). **(D)** Percentages of CD56^–^ PDGFRa^–^ ALP^+^ (circles), of CD56^–^ ALP^–^ PDGFRa^+^ (triangles) and CD56^–^ ALP^–^ PDGFRa^–^ (squares) cells from each patient are reported in the graph. Statistics were performed by unpaired two-tailed *T*-test (*p* > 0.05) for isolation of the CD56^+^ population and one-way ANOVA was performed to assess the MAB, FAP, and IC fractions. Means are indicated per group by a horizontal line.

In both typically developing (TD) children and patients with cerebral palsy (CP), SCs were identified on muscle sections through their co-expression of both PAX7 and CD56 markers (Supplementary Figure 1). The proportion of SCs (recognized as CD56^+^ population) was similar between TD children and patients with CP (29.0 ± 22.6%; *n* = 5 TD samples; 36.5 ± 24.9%; *n* = 15 CP samples; Figures 2A,B). After amplification of the CD56^–^ cells, reasonable amounts of MABs (based on ALP expression: 7.1 ± 6.3%, *n* = 5 TD; 12.4 ± 13.8%, *n* = 8 CP) and FAPs (based on PDGFRa expression: 8.3 ± 15.0%, *n* = 5 TD; 7.4 ± 5.7%, *n* = 8 CP) were isolated with no significant differences between CP and TD for both MABs (CD56^–^ PDGFRa^–^ ALP^+^) and FAPs (CD56^–^ ALP^–^ PDGFRa^+^; Figures 2C,D). Using the CD15 marker, the isolation of FAPs resulted in insufficient cell numbers for the experiments (Supplementary Figure 2B). IC (CD56^–^ ALP^–^ PDGFRa^–^) fractions showed no differences between groups (6.0 ± 3.8%, *n* = 5 TD; 9.2 ± 10.0%, *n* = 8 CP; Figure 2C). Isolated SC-derived populations underwent purity control through FACS analyses resulting in a purity of 93.9 ± 4.3% for CD56^+^ cells (*n* = 10; TD and CP patients) and 98.8 ± 0.5% for CD56^–^ cells (*n* = 4; TD and CP patients) (Supplementary Figure 2A).

### Myogenic Features of Satellite Cell-Derived Progenitors

**FIGURE 3:**
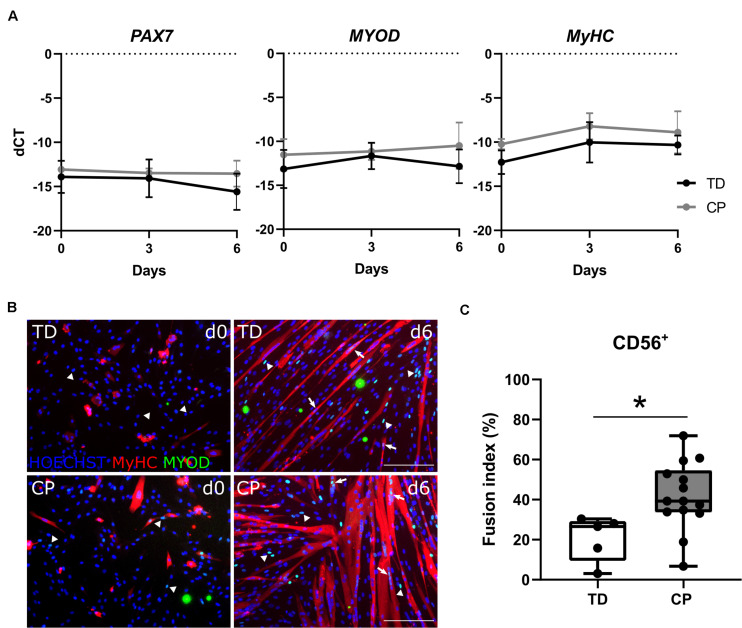
Satellite cell-derived progenitor myogenic differentiation. **(A)** RT-qPCR analysis on SC-derived progenitors from TD children and patients with CP for the expression of *PAX7, MYOD* and *MyHC* genes normalized to β*-ACTIN* gene, at days 0, 3, and 6 during myogenic differentiation. Mean and SD are reported (*n* = 4 for each group). **(B)** Representative immunofluorescence (IF) from SC-derived progenitors of a TD child and a CP patient at days 0 and 6 of myogenic differentiation. MYOD^+^ nuclei (green) are highlighted by arrows when included into myotubes and by arrowheads if not yet fused; MyHC (red) and nuclei are counterstained by HOECHST (blue). Scale bar: 200 μm. **(C)** Fusion index (FI) values are represented by boxplots and dots represent individual subjects (TD: *n* = 5; CP: *n* = 14; *p < 0.05). Statistics were performed by unpaired two-tailed *T*-test and two-way ANOVA (*p* > 0.05).

Analysis by RT-qPCR of the main transcription factors of adult myogenesis, such as *PAX7* and *MYOD*, or a marker of myogenic differentiation, such as *MYOSIN HEAVY CHAIN (MyHC)*, did not show any differences in expression levels during myotube formation (day 0, 3 and 6) between TD children and CP patients (*n* = 4; Figure 3A). The identity of these cells was reconfirmed by immunofluorescence (IF) showing CD56 expression still at passage 7–8 *in vitro*, associated with the nuclear expression of MYOD (Supplementary Figure 3A). The expression of MYOD was shown during the entire differentiation period, during a time course from day 0, 3 to 6 of skeletal muscle induction (Supplementary Figure 3B). Accordingly, SC-derived progenitors of both TD children and CP patients led to varying levels of protein expression for MYOD (Figure 3B, day 0; arrows and arrowheads) and consequently for MyHC, upon differentiation (Figure 3B, day 6). Fusion index (FI) based on MyHC expression was significantly higher in CP (41.7 ± 17.0%, *n* = 14) in comparison to FI in TD children (20.8 ± 11.4%, *n* = 5; *p* < 0.05; Figure 3C). No link between the SC-derived progenitor fusion index with GMFCS level, age or previous botulinum toxin treatments was found (for BTX, see Table 1 for number of patients, dose and conditions). However, our dataset based on these single conditions is probably too limited to lead to robust conclusions (Supplementary Figures 4A–C). Analogously, western blot analysis for MyHC confirmed this heterogeneity, although no clear differences were detected between the two groups, most likely due to the low sample size (*n* = 2 TD; *n* = 3 CP; Supplementary Figures 4D,E).

### Characterization of Mesoangioblasts

**FIGURE 4:**
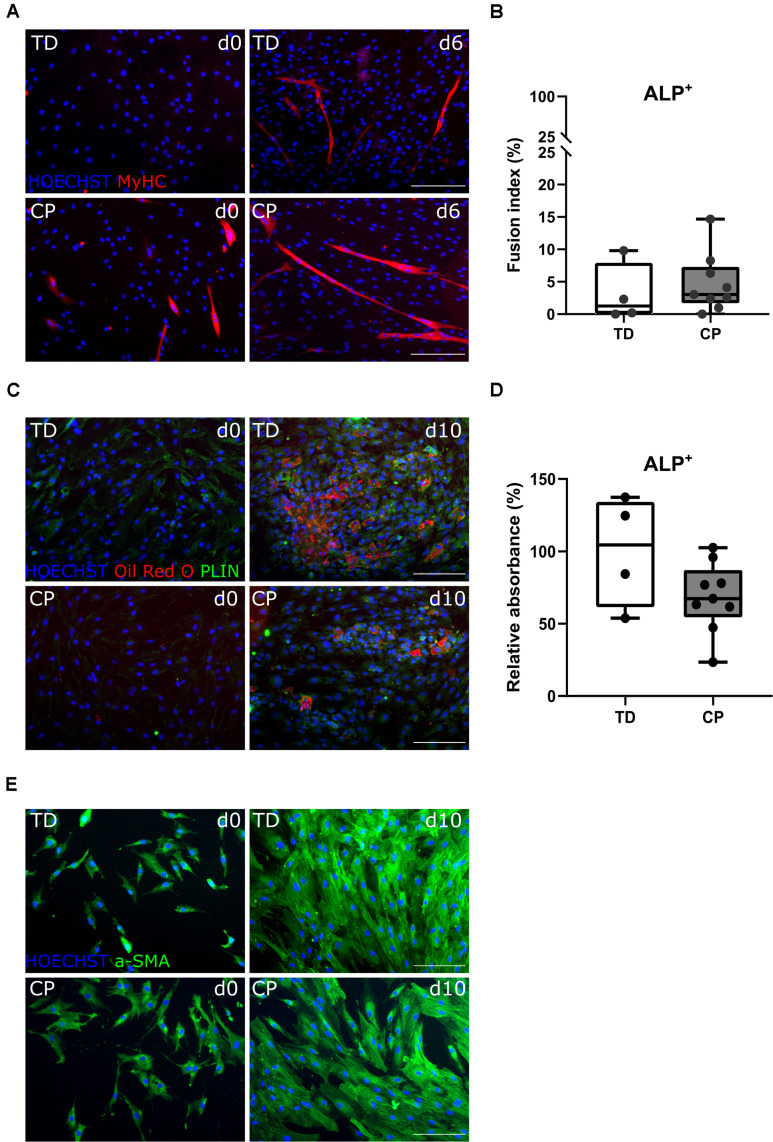
Differentiation assays to characterize the mesoangioblast population. **(A)** Representative IF images from MABs for a TD child and a patient with CP at days 0 and 6 of myogenic differentiation. MyHC (red); nuclei are counterstained by HOECHST (blue). **(B)** FI for TD children (*n* = 4) and CP patients (*n* = 9) is shown in boxplots, dots represent individual subjects. **(C)** Representative IF images of MABs from TD and CP children at days 0 and 10 of adipogenic differentiation. Oil Red O (red); PERILIPIN (PLIN; green); nuclei are counterstained by HOECHST (blue). **(D)** Oil Red O quantification at day 10 of adipose differentiation was measured and normalized to TD samples (TD: *n* = 4; CP: *n* = 9). Data are shown in boxplot graphs, individual subjects are represented by dots. Statistics were performed with unpaired two-tailed Student’s *T*-test (*p* > 0.05). **(E)** Representative IF images at days 0 and 10 of smooth muscle differentiation of MABs from TD and CP children. α SMOOTH MUSCLE ACTIN (a-SMA; green) and nuclei are counterstained by HOECHST (blue). Scale bars are 200 μm.

After amplification of the CD56^–^ population, in a second cell sorting, we extracted CD56^–^ PDGFRa^–^ ALP^+^ cells, hereby referred to as MABs. They differentiated toward myotubes with fusion index values of 3.1 ± 4.6% (*n* = 4 TD) and 4.7 ± 4.5% (*n* = 9 CP; Figures 4A,B). Myogenic potential based on fusion index did not differ significantly between the two groups. Adipogenic potential of this population was confirmed by Oil Red O staining and PERILIPIN expression (PLIN; Figure 4C; Dellavalle et al., 2007). Quantification of Oil Red O within CP adipocytes was expressed as a percentage of TD samples (100 ± 38.2%, *n* = 4 TD; 68.5 ± 24.0%, *n* = 9 CP) and did not show differences (Figure 4D). To confirm the multipotent character of MABs, smooth muscle differentiation assays were performed on a representative fraction of samples (*n* = 3 per group). Based on IF staining, higher levels of α SMOOTH MUSCLE ACTIN (a-SMA) were expressed after 10 days of differentiation in comparison to day 0 (Figure 4E).

### Differentiation Potential of Fibro-Adipogenic Progenitors

**FIGURE 5:**
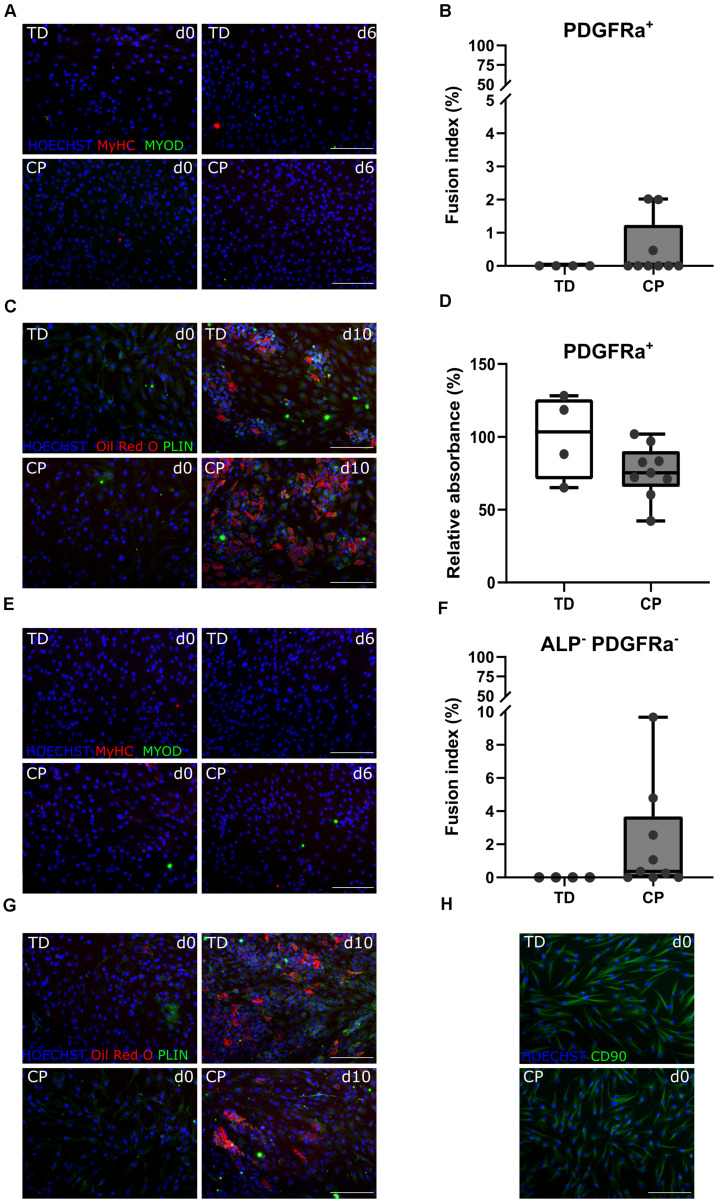
Characterization fibro-adipogenic progenitors and interstitial cells. **(A)** Representative IF images of FAPs for a TD child and a patient with CP at days 0 and 6 of myogenic differentiation. MYOD (green); MyHC (red); nuclei are counterstained by HOECHST (blue). **(B)** Fusion Index (FI) for TD children (*n* = 4) and CP patients (*n* = 9) is shown in boxplots, dots represent individual subjects. **(C)** Representative IF images of FAPs from TD and CP children at days 0 and 10 of adipogenic differentiation. Oil Red O (red); PLIN (green); nuclei are counterstained by HOECHST (blue). **(D)** Oil Red O quantification at day 10 of adipose differentiation was measured and normalized to TD samples (TD: *n* = 4; CP: *n* = 9). Data are shown in boxplot graphs, individual subjects are represented by dots. **(E)** Representative IF images of ICs from TD children and patients with CP at days 0 and 6 of myogenic differentiation. MYOD (green); MyHC (red); nuclei are counterstained by HOECHST (blue). **(F)** FI for TD children (*n* = 4) and CP patients (*n* = 9) is shown in boxplots, dots represent individual subjects. **(G)** Representative IF images of ICs from TD and children with CP at days 0 and 10 of adipogenic differentiation. Oil Red O (red); PLIN (green); nuclei are counterstained by HOECHST (blue). **(H)** Representative IF images of ICs from both TD children and patients with CP stained for CD90 (green); nuclei are counterstained by HOECHST (blue). Scale bars are 200 μm. Statistics were performed using an unpaired two-tailed Student’s *T*-test (*p* > 0.05).

The CD56^–^ ALP^–^ PDGFRa^+^ population, also referred to as FAPs, showed very limited myogenic capacity based on IF staining for MYOD and MyHC, after a 6-day myotube differentiation, for TD children and patients with CP (Figure 5A). Fusion index values for TD children (0.0 ± 0.0%, *n* = 4) as well as for children with CP (0.5 ± 0.9%, *n* = 9) were very low and not significantly different between both groups (Figure 5B). Adipogenic potential of this population was tested and confirmed by Oil Red O and PLIN staining (Figure 5C). Quantification of Oil Red O within the adipocytes from CP samples (76.2 ± 18.1%, *n* = 9), compared to the absorbance observed in TD samples (100 ± 28.9%, *n* = 4), did not show any significant differences (Figure 5D).

### Characterization of Interstitial Cells

After isolation of the most well-known muscle stem cell-derived progenitors, the remaining myogenic capacity in the ICs (CD56^–^ ALP^–^ PDGFRa^–^) showed a low myogenic potential, based on IF staining for MYOD and MyHC, as well as by fusion index (Figures 5E,F), in both groups. IC-derived myotubes were totally absent in TD children (0 ± 0.0%, *n* = 4), or sporadically present in patients with CP resulting in a low and not significantly different fusion index (2.1 ± 3.1%, *n* = 9). ICs also showed high adipogenic differentiation potential based on Oil Red O staining and expression of PLIN (Figure 5G), without any significant differences for CP with respect to TD values. This cell population was indeed characterized in cells from both origins by the expression of CD90 (Figure 5H), a marker usually used for fibroblasts (Kisselbach et al., 2009).

### Qualitative Differences in Myotubes From Satellite Cells Between TD Children and Patients With CP

**FIGURE 6:**
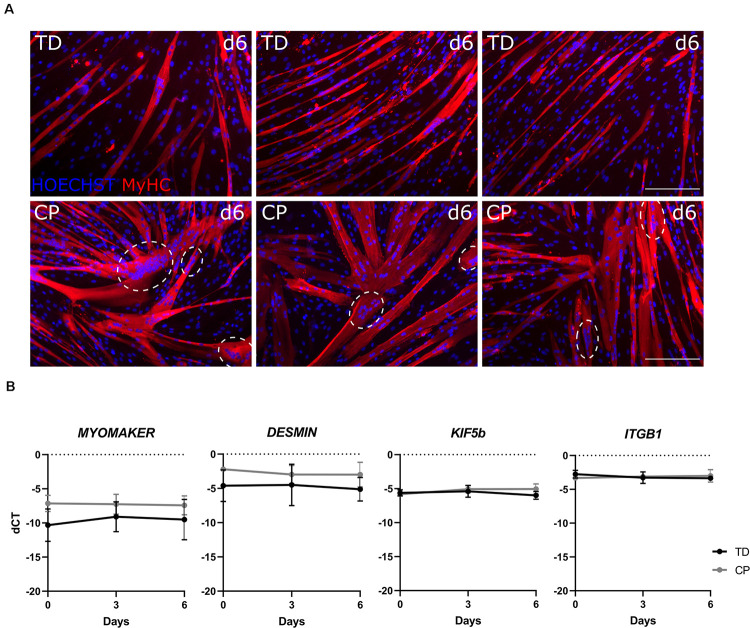
Altered differentiation of satellite cell-derived progenitors. **(A)** IF images of SC-derived progenitors from three representative TD children (top panels) and three representative patients with CP (lower panels) after 6 days of myogenic differentiation. MyHC (red); nuclei are counterstained by HOECHST (blue). Dotted circles indicate co-localization of the nuclei. Scale bar is 200 μm. **(B)** RT-qPCR analysis on SC-derived progenitors from TD children and CP patients for *MYOMAKER, DESMIN, KIF5b*, and *ITGB1* expression normalized to β*-ACTIN* expression during myogenic differentiation at days 0, 3, and 6 (*n* = 4 for each group). Data are expressed as mean and SD. Two-way ANOVA was performed (*p* > 0.05).

We observed SC-derived progenitor myogenic differentiation alterations in mature myotubes based on IF staining for MyHC. IF images of differentiated SC-derived progenitors from three representative TD children showed rather long and thin myotubes, while the ones from three representative patients with CP seemed to be larger and less characterized by the usual tubular shape (Figure 6A). Additionally, in myotubes from CP patients, we noticed the nuclear tendency to co-localize in big clusters, which was not observed in myotubes from TD children. Transcript levels for multiple genes involved in fusion and nuclear migration did not show any significant differences between TD children and patients with CP (*n* = 4 per group; Figure 6B).

## Discussion

For the first time, this study applied the muscle microbiopsy technique for the successful isolation, culture, amplification and examination of different adult muscle stem cell-derived progenitors in young TDs and children with CP. The muscle microbiopsy was very well tolerated and proved to be safe in young children. Moreover, it delivered a sufficient amount of material to investigate the muscle tissue of very young subjects. An important advantage of the microbiopsy is that this technique more easily allows including young children and obtaining muscle material by a less invasive method, despite the required *in vitro* passages for all the assessments. Other groups have previously successfully explored the microbiopsy technique in *Vastus Lateralis* of adult patients with chronic obstructive pulmonary disease. These studies reported on the feasibility and efficacy of this minimally invasive technique, which appeared more comfortable compared with the commonly applied Bergström technique (Magistris et al., 1998; Hayot et al., 2005; Pomiès et al., 2015; Townsend et al., 2016). In the current study, the optimal location for sample collection was defined using ultrasonography. Moreover, to minimalize heterogeneity, the standardized protocol relied on the same experienced team of surgeons and researchers to collect and handle muscle microbiopsies.

Based on the limited amount of material obtained from the muscle microbiopsy, we and other groups confirmed the impossibility to directly isolate sufficient numbers of stem cells for further studies after direct enzymatic digestion of microbiopsies (data not shown; Ceusters et al., 2017). Furthermore, this technique avoids applying an aggressive approach of tissue digestion that could damage the cell surface receptors and which could interfere with cell integrity, identity and function (Ceusters et al., 2017). Therefore, we opted for the explant technique, which allows progenitor cell migration out from the tissue and stimulates proliferation to reach adequate cell numbers for isolation (Cusella-De Angelis et al., 1994; Messina et al., 2004; Morosetti et al., 2006; Tonlorenzi et al., 2007). Cells were amplified and different cell populations could be isolated by FACS, based on the expression of specific markers used in literature (Rotini et al., 2018; Tey et al., 2019). The co-localization for CD56 and PAX7 was verified in muscle slides from TD and CP patients. However, SC-derived progenitors, when amplified *in vitro*, have been reported to lose the expression of PAX7. Indeed, recent data report that SC-levels of PAX7, detectable at 24 h after extraction, dramatically decrease at passage 4 *in vitro* (Romagnoli et al., 2020). Therefore, to confirm the identity of SC-derived population, we showed the expression of CD56 marker together with MYOD, the master-gene for muscle development. Moreover, MYOD is continuously present in the cells during the entire differentiation period (Yin et al., 2013). Although the number of the cells obtained was sufficient to proceed with the sorting, we applied a serial sorting, in order to pick single positive cells and easily avoid double stained cells. After amplification of the CD56^–^ population, we successfully isolated MABs and FAPs. However, the percentages of isolated fractions were very variable between and within the sample groups. In future analyses, immunohistochemistry assessments are planned on additional muscle samples obtained from the same subject in order to correlate these cell fractions to the *in vivo* condition of the muscle (Townsend et al., 2016). These further assessments would allow us to directly characterize these stem cells from freshly digested samples, in order to compare the proportion of amplified cells extracted with our procedure with the real cell percentage in the muscle. Two additional challenges related to the sample quality need to be assessed. Firstly, with this technique the obtained muscle volume was small due to the young age of the children. Secondly, altered muscle features in patients with CP, such as higher collagen content, can decrease the amount of muscle material able to give rise to cells (Booth et al., 2001; Tisha et al., 2019).

Finally, other factors could alter the number of the cells obtained, such as the mechanical load of muscle tissue shortly before the microbiopsy session as shown in adult subjects (Mackey et al., 2007). By contrast, the only study available in young athletes reported no statistical differences in the number of SCs after exercise (Metaxas et al., 2014), although the absence of a control group precludes to run constructive comparisons. In our subject database, no differences in duration of physiotherapy were reported among the CP patient subgroups based on GMFCS levels. A more precise survey regarding the physical activity level prior to the biopsy may help to avoid variations due to exercise-induced activation of SCs and possibly of inflammation-activated cells. Nevertheless, our workflow was successful in providing enough cells in all subjects irrespectively of the aforementioned challenges and of the pathological involvement.

SC-derived progenitors from both TD children and patients with CP children expressed *PAX7*, *MYOD*, and *MyHC* transcripts during myogenic differentiation. Protein levels of CD56 and MYOD confirmed the identity of these cells also after several passages. After 6 days of myogenic differentiation, SC-derived progenitor cultures showed the highest level of maturation, based on myotube formation and MyHC staining. Unfortunately, after this time point, the risk of detaching myotubes due to stress and potential *in vitro* contractions increased in both CP and TD. Therefore, we performed all the analyses at day 0, 3 and 6, in order to show that the cells from TD and CP children maintain MYOD expression during all the differentiation period. Fusion index showed that SC-derived progenitors are able to engage in myogenic differentiation *in vitro* and those of patients with CP differentiate to a higher extent compared to the one of TD children. We found a strong effect size (1.45) when fusion index values were compared between SC-derived progenitors from TD children and patients with CP, indicating that our sample size was sufficient to reveal a significantly different fusion index. These results confirmed the only previous finding on myogenic differentiation reported for children with CP (Domenighetti et al., 2018). It is possible that inflammation or other environmental cues in the CP muscle are favoring their activation (Costamagna et al., 2015). However, possible associations between fusion index and GMFCS level, age or previous BTX treatments were not found in the current study groups. This might be related to the small sample size per group. Another parameter that could influence the differentiation potential of these stem cell-derived progenitors is the number of passages. In our case, for all the included participants, passage numbers were very stable and comparable per cell type, due to the standardized protocol used. For one TD case, we isolated a very small SC-derived progenitor fraction, resulting in a higher passage number that consequently determined the lowest fusion index reported in Figure 3. Our finding of higher SC-derived progenitor fusion index in myotubes from CP compared to TD children are in contrast to previous results, in which data from CP patients showed fewer thin spindly myotubes with lower fusion indexes (Domenighetti et al., 2018). Discrepancies between the results of the current study and those of Domenighetti and colleagues could be attributed to several differences in the techniques used or in the conditions applied. These include the heterogeneity of the patients with CP and the lack of age-matched TD children. Domenighetti et al. (2018) included 8 children with an average age of 9 ± 4 years, and 15 TD children with an average age of 15 ± 3 years, while the current study focused mainly on younger children with lower variances in age (6.3 ± 2.0 years old, *n* = 15) compared to age-matched TD children (5.1 ± 1.4 years old, *n* = 5). Furthermore, initial patient material (amount of tissue, autograft or microbiopsy) and the specific target muscle (*hamstring* muscles compared to *Medial Gastrocnemius*, distal portion compared to mid-belly region) were different. The conditions of culture and differentiation were also different between the two studies, such as the growing media (enriched with hFGF or not) and the differentiation media (enriched with insulin or not), the time of myotube differentiation (42 h or 6 days) and the coating surface favoring cell adherence (0.5% gelatin or 0.1% collagen). Nevertheless, the contrasting results highlight the complexity and the heterogeneity of CP. We do not know whether the most prominent difference is related to patient characteristics, such as age, GMFCS level, uni or bilateral involvement, BTX history etc. or to differences in study design or a synergic effect of both. Further research will be needed to better understand the complexity and heterogeneous nature of CP pathology.

Additionally, after 6 days of myogenic differentiation, based on IF staining for MyHC, we report thicker and less spindle shaped myotubes in the SC-derived progenitor cultures from multiple patients with CP in comparison to those from TD children. Further targeted western blot analysis will better quantify these observational differences, as we have already shown the feasibility of this technique on few samples (Supplementary Figures 4D,E). We also observed more pronounced grouped nuclei within the MyHC^+^ areas. Interestingly, this phenomenon was mainly observed in CP patients with GMFCS level II or III. Further analysis will confirm whether this nuclear co-localization is a phenomenon also occurring in longitudinal sections of muscle tissue from CP and whether this can correlate with the degree of pathological involvement. Along the same lines, the early phases of fusion process in muscle regeneration are highly complex, involving crosstalk of many signaling pathways and the coordination of multiple transcription factors. We explored some indicators of the observed alterations in SC-derived myotube morphology based on gene expression. Because the process of myoblast fusion has mainly been studied and understood in murine SCs, we searched for human homologs or correspondent human equivalents for the many genes involved, such as *MYOMAKER, DESMIN, KIF5b, and ITGB1* (Millay et al., 2013; Kim et al., 2015; Quinn et al., 2017; Bi et al., 2018). Additionally, after fusion, some of these genes are also implicated in nuclear alignment, which is an important process during maturation (Wang et al., 2013; Cadot et al., 2015; Gache et al., 2017; Starr, 2017). Defects in nuclear positioning can lead to centronuclear myopathies with clinical representations such as muscle weakness and atrophy (Jungbluth et al., 2008; Romero, 2010). The fact that we did not see significant differences in the preliminarily assessed genes does not exclude that other regulators than the ones we examined could be altered. Further analyses will be helpful to better shed light on CP SC-derived progenitor alterations.

Currently, there is a lack of knowledge concerning other stem cell types playing a role in postnatal development and remodeling processes in CP. In accordance with the literature, the vessel-associated MABs withhold myogenic potential, albeit lower than the one of SCs (Dellavalle et al., 2007; Tey et al., 2019). Differently from SCs, MABs maintain a higher migration potential, a feature that makes them appealing for cell transplantation (Cossu et al., 2015). Here, MABs and FAPs were characterized by their myogenic and adipogenic potency *in vitro* and did not seem to differ significantly between TD children and CP patients. Moreover, reasonable numbers of FAPs were isolated based on PDGFRa expression. However, these data should be taken with care due to the explorative nature of the current analysis and because other studies isolating FAPs from human young muscle tissue are still missing. Nevertheless, two different markers, such as PDGFRa and CD15, have been described as valid markers for this population (Uezumi et al., 2010; Arrighi et al., 2015; Madaro et al., 2018). In our conditions, CD15^+^ population showed lower percentages than the PDGFRa^+^ population and further studies are needed to better document which of these markers better suits the population studied in adults or young patients. Characterization of these cells was obtained through differentiation potential. FAPs cells showed none to very limited number of myotubes upon myogenic induction, which is characteristic of this cell type (Joe et al., 2010; Uezumi et al., 2010, 2014). Random purity checks showed high efficacy in our conditions, excluding possible limitation of the FACS technique. Further analyses will help to define whether MABs and FAPs can play a role in the altered panorama of muscle stem cells, especially referring to the aberrant fat deposition and to the secretion of cytokines recruiting inflammatory cells and promoting migration (Camps et al., 2019). The IC-derived-population was assessed for both myogenic and adipogenic capacity and stained for the presence of fibroblast marker CD90. Based on myogenic and adipogenic potency from these cells, no differences were found between data from TD children and patients with CP, suggesting that this cell population is most probably not altered by the pathology. Obviously, many other cells, such as macrophages and fibroblasts could play a role in the CP pathology and their implication should not be neglected. Those cells would be interesting targets for future research (Peterson et al., 2012; Lemos et al., 2015; Deyhle and Hyldahl, 2018; Von Walden et al., 2018; Wang et al., 2019; Sorensen et al., 2019). Similarly, the relevance of other muscle stem cell populations not investigated in the current study needs to be considered in future studies. In particular, possible interactions between these different cell types need to be addressed in future research, with a larger sample size and more exhaustive techniques.

## Conclusion

In conclusion, this study confirmed the use of minimally invasive and well-tolerated muscle microbiopsy as a feasible approach to obtain muscle samples from very young children. This allows performing extensive analyses at the microscopic level. Furthermore, we showed that myogenic differentiation of SC-derived progenitors, based on fusion index, is increased in young patients with CP in comparison to TD children. Interestingly, we reported alterations in myotube morphology and nuclear localization with a still qualitative and descriptive approach. Further studies are necessary to overcome unsolved questions regarding the muscle pathology associated with CP, which were partially unraveled by cell culture analyses. Overall, the current results strengthen the hypothesis of muscle stem cell involvement in CP pathology. Finally, by highlighting the relevance of the muscle microbiopsy technique as a novel approach for collecting muscle tissue in young patients with CP, we hope to pave the way for research in very young children, which may lead to a better understanding of the onset and development of CP pathophysiology.

## Data Availability Statement

The original contributions presented in the study are included in the article/Supplementary Material, further inquiries can be directed to the corresponding author.

## Ethics Statement

The studies involving human participants were reviewed and approved by the Ethical Committee of the University Hospitals of Leuven, Belgium (S61110 and S62645). Written informed consent to participate in this study was provided by the participants’ legal guardian/next of kin.

## Author Contributions

All authors conceived and discussed experiments, read and approved the final version of the manuscript. In particular, AC and SP are pediatric orthopedic surgeons, who obtained the muscle microbiopsies and together with ND were responsible for patient recruitment. MC, RD, JD, and DC performed the experiments. MC, ND, RD, JD, and DC analyzed the data and prepared the figures. MC and DC wrote the manuscript. GG-R, KM, GW, KD, and MS edited and revised the manuscript.

## Conflict of Interest

The authors declare that the research was conducted in the absence of any commercial or financial relationships that could be construed as a potential conflict of interest.
