# Supplementary figures and images for: Muscle Microbiopsy to Delineate Stem Cell Involvement in Young Patients: A Novel Approach for Children With Cerebral Palsy
Source: Front Physiol. 2020 Aug 6;11:945. doi: 10.3389/fphys.2020.00945 (PMC7424076; doi:10.3389/fphys.2020.00945)

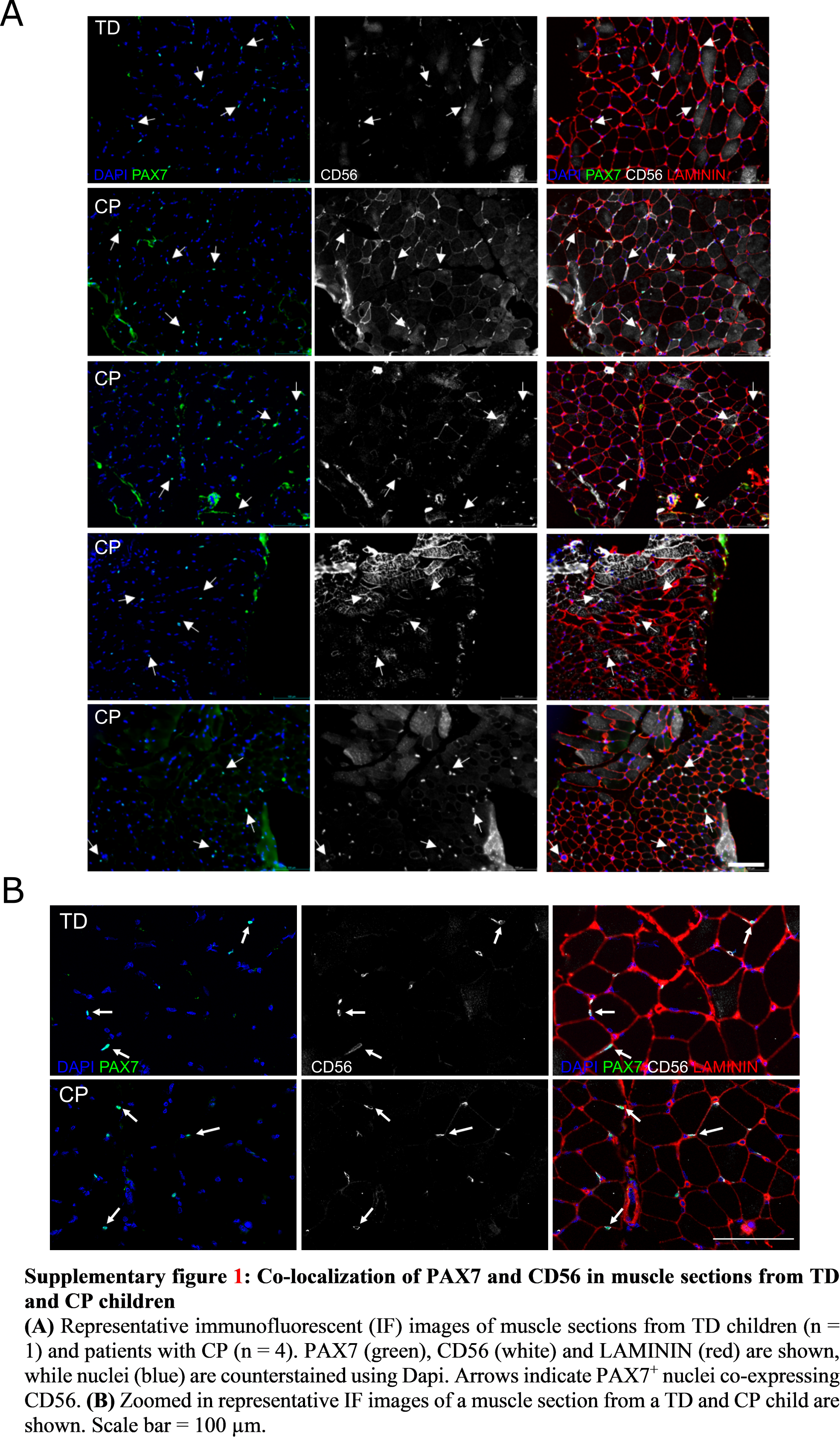

Supplement: Supplementary file 1 [file Image_1.tiff]

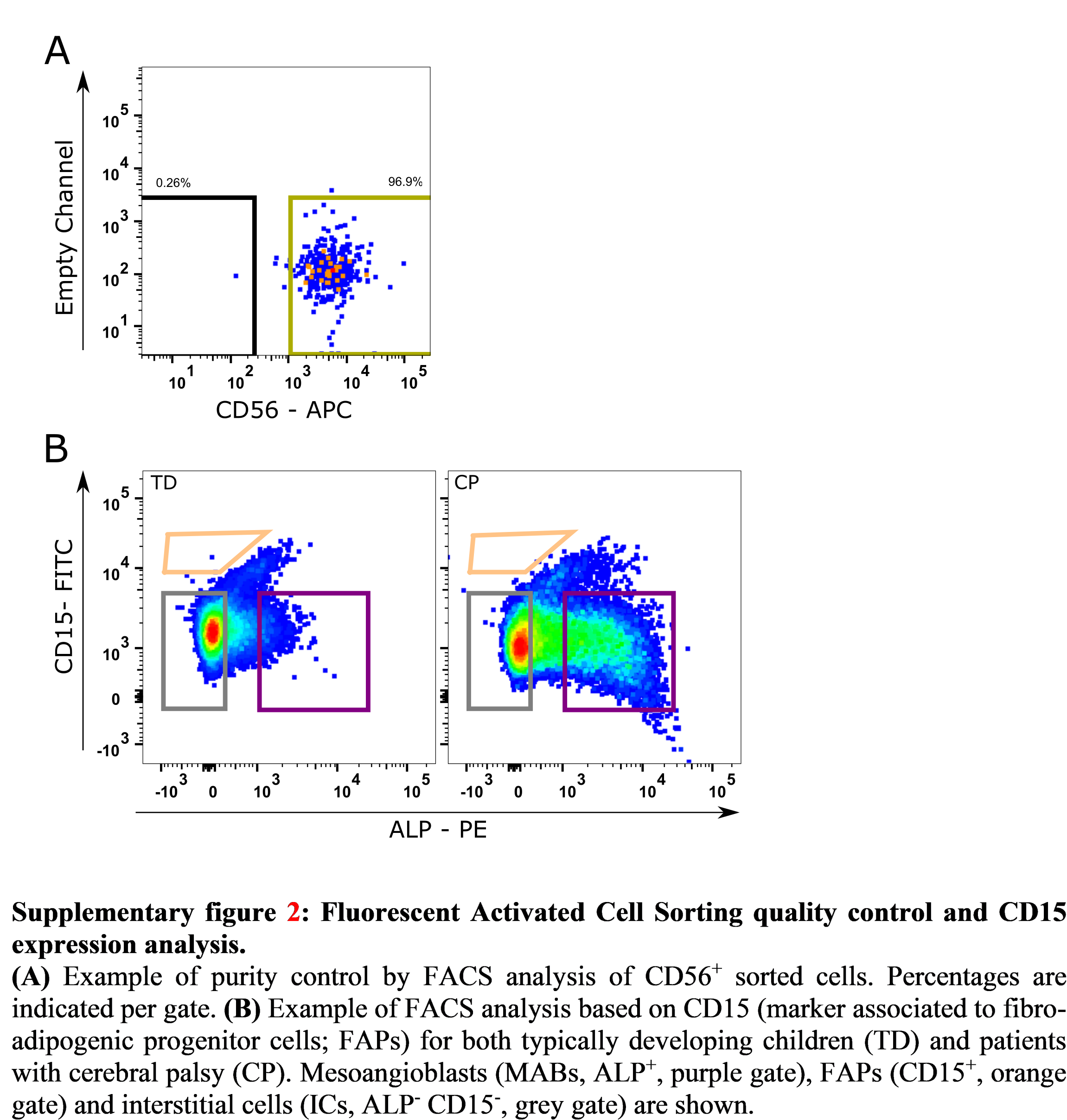

Supplement: Supplementary file 2 [file Image_2.TIF]

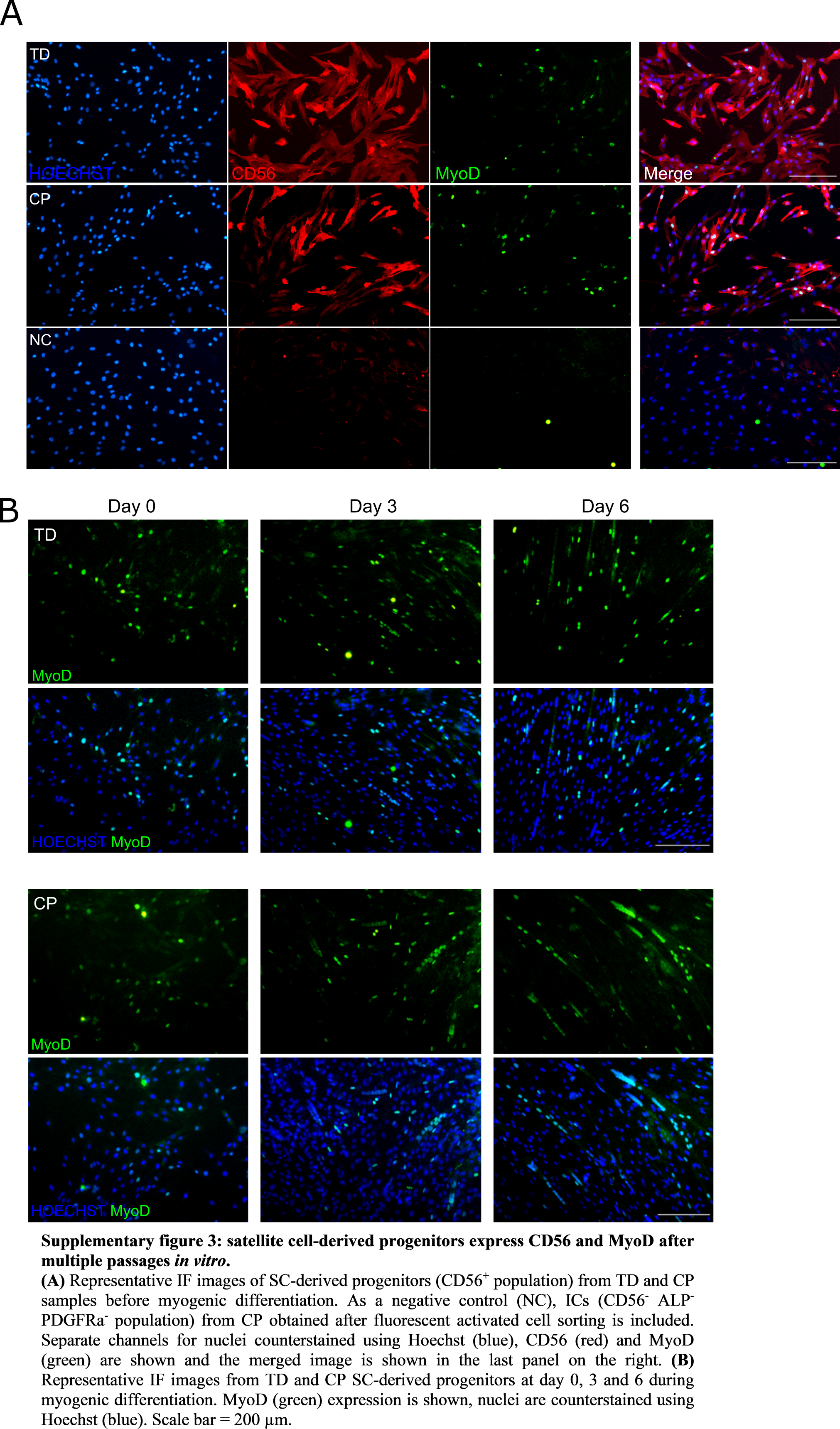

Supplement: Supplementary file 3 [file Image_3.TIF]

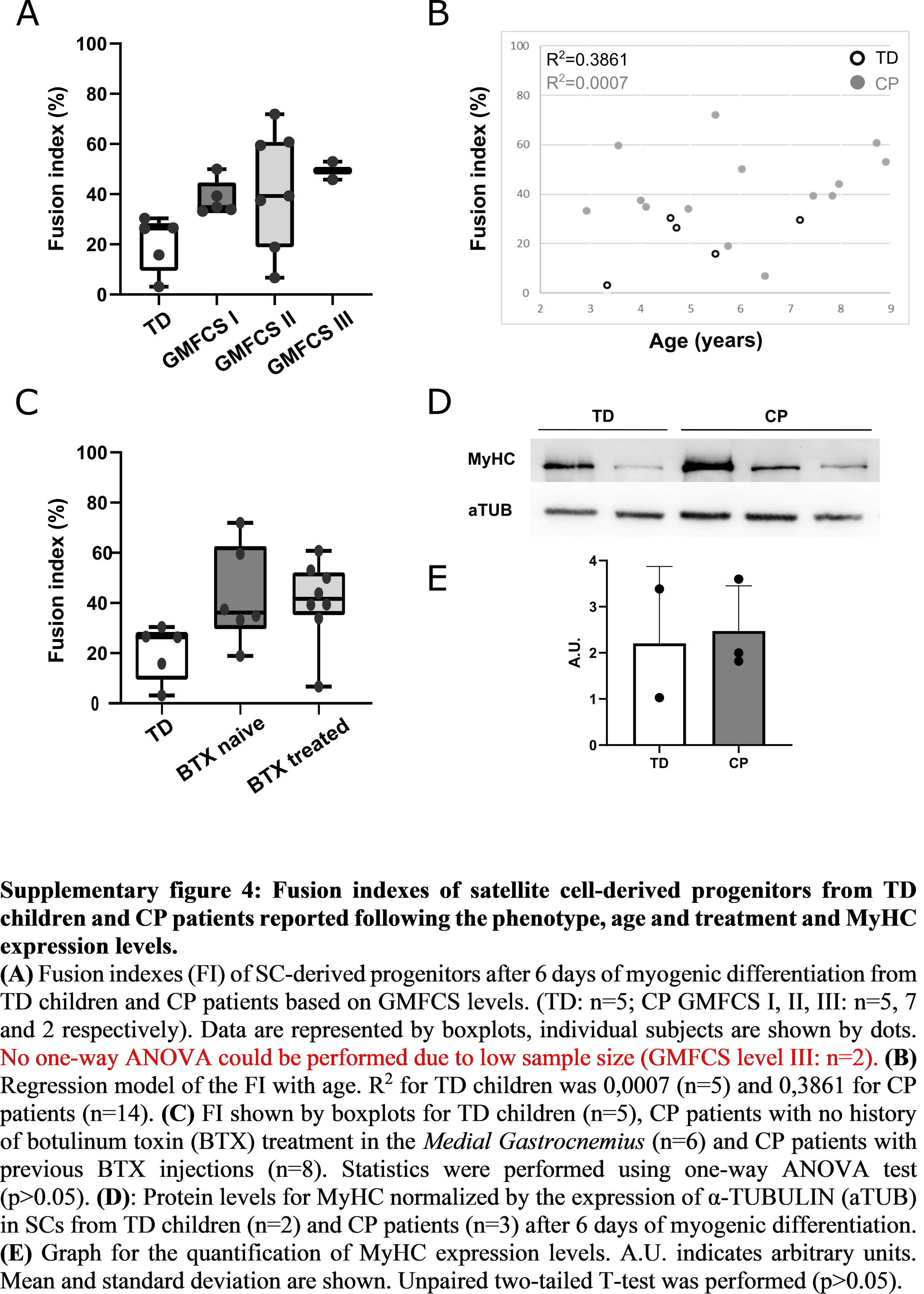

Supplement: Supplementary file 4 [file Image_4.TIF]
